# Connected Elbow Exoskeleton System for Rehabilitation Training Based on Virtual Reality and Context-Aware

**DOI:** 10.3390/s20030858

**Published:** 2020-02-06

**Authors:** Daniel H. de la Iglesia, André Sales Mendes, Gabriel Villarrubia González, Diego M. Jiménez-Bravo, Juan F. de Paz Santana

**Affiliations:** 1 Expert Systems and Applications Lab, Faculty of Science, University of Salamanca, Plaza de los Caídos s/n, 37002 Salamanca, Spain; andremendes@usal.es (A.S.M.); gvg@usal.es (G.V.G.); dmjimenez@usal.es (D.M.J.-B.); fcofds@usal.es (J.F.d.P.S.); 2 Facultad de Informática, Universidad Pontificia de Salamanca, C/Compañía 5, 37002 Salamanca, Spain

**Keywords:** exoskeleton, elbow rehabilitation, virtual reality, edge computing

## Abstract

Traditional physiotherapy rehabilitation systems are evolving into more advanced systems based on exoskeleton systems and Virtual Reality (VR) environments that enhance and improve rehabilitation techniques and physical exercise. In addition, due to current connected systems and paradigms such as the Internet of Things (IoT) or Ambient Intelligent (AmI) systems, it is possible to design and develop advanced, effective, and low-cost medical tools that patients may have in their homes. This article presents a low-cost exoskeleton for the elbow that is connected to a Context-Aware architecture and thanks to a VR system the patient can perform rehabilitation exercises in an interactive way. The integration of virtual reality technology in rehabilitation exercises provides an intensive, repetitive and task-oriented capacity to improve patient motivation and reduce work on medical professionals. One of the system highlights is the intelligent ability to generate new exercises, monitor the exercises performed by users in search of progress or possible problems and the dynamic modification of the exercises characteristics. The platform also allows the incorporation of commercial medical sensors capable of collecting valuable information for greater accuracy in the diagnosis and evolution of patients. A case study with real patients with promising results has been carried out.

## 1. Introduction

Currently, there are more than 890 million people with chronic diseases worldwide [1]. It is estimated that 25% of these patients could benefit immediately from solutions for monitoring their health from home [2]; another 50% would benefit from the integration in their mobile phones or other devices of existing medical resources [3,4]. In Europe, there is a large group of people suffering from some type of chronic diseases, such as diabetes or cardiovascular diseases. To these data, we must add all those patients who suffer from some type of mobility problem or have suffered an accident or limb fracture. Considering the advances made in technologies such as the Internet of Things, e-health, or the mbient Assisted Living, monitoring health and improving patients from their own homes is now an unquestionable reality. Ambient Intelligence (AmI) is a paradigm that seeks to build intelligent models capable of adapting to the user’s environment, responding in an appropriate way to the needs detected and with the ability to sensor both physical and human assets [5]. In recent years, numerous scientific works related to this important area have proliferated. A large part of these works is focused on the use of different medical sensors for remote monitoring, disease monitoring or early warning of new pathologies. There are fewer studies that investigate how the technologies listed above can help users to rehabilitate certain minor pathologies in the comfort of their home without going to medical centers. This work tries to contribute a new practical case of AmI technology applied to medical domestic environments for the active rehabilitation of a patient.

One of these cases is physiotherapeutic rehabilitation patients who, through current technological advances, could benefit from adapted, effective and easy-to-use rehabilitation systems from their own home. The exoskeleton-based rehabilitation systems available today for upper limbs are complex systems, difficult to install on the arm, and difficult to use. In addition, they have a heavy size that prevents the arm from moving naturally, making rehabilitation tasks difficult. Another added problem lies in the price of these devices, which causes that only the institutions and health centers can acquire them. The user does not have the possibility of carrying out rehabilitation exercises from the comfort of their home, they are also forced to go periodically to the physiotherapy center, which has the necessary equipment. Traditional rehabilitation systems also have a lack of motivation problem for patients, the vast majority of the exercises are based on repetition and this makes the sessions become monotonous and not very stimulating.

The main objective of this work is to design a rehabilitation device based on an exoskeleton for the elbow of a degree of freedom (one DOF) connected to a virtual reality system called EXOMedical. This Virtual Reality (VR) system is specially designed for working immersively with the user, controlling the motor of the exoskeleton, and measuring the force exerted by the patient. In addition, a Context-Aware architecture [6] has been designed to control all the data generated by the exoskeleton, detecting user progress and identifying possible problems in advance. In addition, it will be able to generate new exercises adapted to progress and patient ability. This architecture will also have the assistance of the medical professional in charge of rehabilitation, which can continuously monitor its evolution, allowing personalized follow-up on each patient.

Currently, the rehabilitation process is carried out in a hospital or in specialized physiotherapy centers with the help and supervision of a professional in that area. The professional must perform the work of evaluation or triage as well as the monitoring and performance of the corresponding therapy. Due to the high number of patients, it may lead to the need for a large number of professionals to carry out all these activities and attend them correctly. In the same way, it is difficult to quantify the improvement or evolution of patients since there is currently no way to quantify them and the subjective assessment of an expert must be used. The developed system allows a quantitative measure of the evolution and capabilities of the patient over the Context-Aware architecture that has historical data on progress individually and in detail of the entire recovery. These data will serve the system to analytically measure the patient’s recovery, as well as the generation of new exercises adapted to that evolution. Although these processes are normally carried out in physiotherapy centers, the user can use it in a more comfortable place, such as in their own home, transmitting the data of their evolution to the platform, generating a more favorable and collaborative attitude on the patient part. As for chronic patients, this device allows an increasement in their quality of life by assisting in weightlifting. For example, a person who has suffered a car accident in which his arm has been damaged, or someone who has suffered a work accident or a stroke, can perform daily tasks such as moving weights of up to 15 kg without letting the effort fall on your body.

This article is structured as follows: Section 2 reviews the current state of the art; Section 3 describes the proposed system in detail; Section 4 introduces the case study with real patients; Finally, conclusions drawn from the work are outlined in Section 5.

## 2. Background

In the current literature, it is possible to find articles that address the use of robotic exoskeleton systems for the recovery of patients with mobility problems. The vast majority of these works are focused on the recovery of injuries and problems of the upper limbs. There are other works that focus on the study of injuries from other areas such as the ankle [7,8,9]. Other works focused on one of the most important joints of the lower extremities such as the knee [10,11,12,13,14,15]. In all these works, the use of robotic exoskeleton systems is another part of the traditional physiotherapeutic rehabilitation that is advised by an expert and is performed in a traditional clinical setting.

In the works that address the recovery of the joints in the upper extremities, there are different projects that seek to provide significant progress [16]. These works combine, like this article, the use of a robotic exoskeleton, with virtual reality technology and environments that allow patients to develop the exercises in a more dynamic and interactive way. It is possible to identify, within the upper extremities, those works focused on the rehabilitation of the hand (wrist and fingers), the rehabilitation of the elbow and the integral rehabilitation of the entire upper limb. In the work [17], the authors present a study based on the generation of virtual reality-based interfaces for the rehabilitation of arms and hands, in which the use of optoelectronic sensors such as Leap Motion, Microsoft Kinect and Oculus VR is necessary. These devices are capable of capturing user movements and translating them into digital environments that motivate the user to participate in dynamic rehabilitation sessions. In the case of the studies [18,19], the authors focus their work on the treatment of hand-related problems. The authors of [20] propose a system based on fuzzy logic, virtual environments, and an active hand orthosis operated through servomotors that allow exerting a certain force with which users can perform different strengthening exercises. The combination of flexibility sensors, servomotors, and optoelectric control of the Leap Motion sensor generates an accurate simulation of hand movements in the virtual environment.

The article that addresses the integral physical rehabilitation of the arm, highlights numerous works that use virtual reality techniques as an additional recovery tool. The authors of the paper [21] propose the use of an exoskeleton device with 5 DOF for the integral rehabilitation of the arm using virtual environments during a session with a medical professional. In the article [22], the authors propose an active rehabilitation training system based on virtual reality technology specially designed for patients with upper limb hemiparesis. These authors develop several virtual games to increase the interest of patients when performing the exercises. In the same area, the authors of [23] use an exoskeleton of 5 DOF but making use of immersive environments with virtual reality as well as the work of the authors of [24].

Another researching lines in upper limb rehabilitation devices are stroke patients who require specific rehabilitation to recover effective arm movement. The authors of the work [25], in their pilot study with a stroke patient working with an exoskeleton for the arm, determined that its use was very advantageous with respect to patients following a more traditional recovery. Similarly, the study [26] highlights this type of therapy based on the use of exoskeletons and virtual environments as potentially more beneficial than traditional therapies as a result of a large study with real patients. The authors of [27] implement a modular and reconfigurable exoskeleton, which seeks to reduce costs and size by adopting different therapeutic end effectors for different training movements using a single robot in stroke patients.

As for the works that address the design of Context-Aware architectures and frameworks, it is possible to find a large number of works based on telemonitoring and diseasing monitoring systems [28,29,30,31]. It is less frequent to find works that present this type of architecture focused on rehabilitation exercises for home monitoring, in combination with telemonitoring and e-health. These works include the work of the authors of [32] whose objective is to integrate software architectures with person-computer interfaces to develop context-sensitive systems for telerehabilitation of people from their homes. Another outstanding work is that of the authors of [33] where a Context-Aware framework is proposed and validated for the use of animatronic biofeedback, as a way to potentially increase the compliance of older users with physical rehabilitation exercises performed in home. In this context, animatronic biofeedback involves the use of preprogrammed actions in a robot that are activated in response to certain changes detected in the biomechanical or electrophysiological signals of the users. Another paper presented is [34] where the authors present a model-based approach to the development of telerehabilitation systems through context-aware systems.

It is possible to summarize that there is no work in the current literature that comprehensively addresses the design of a Context-Aware system in combination with a low-cost exoskeleton integrated with a virtual reality environment for performing rehabilitation exercises. The current systems based on exoskeletons are supervised and unconnected systems, without the possibility of sharing the data obtained or generating new exercises dynamically as the device proposed in this work.

## 3. Proposed System

This section presents the system proposed in this work. Firstly, it is described in a connected hardware device that will serve as a rehabilitation exoskeleton for system patients. Next, the architecture based on Context-Aware technology for monitoring and control of the rehabilitation system is described. Finally, the virtual reality system that will connect to the hardware device and Context-Aware architecture and whose purpose is to provide an immersive user interface to make physical exercises is analyzed.

### 3.1. One DOF Exoskeleton for Elbow

**Figure 1:**
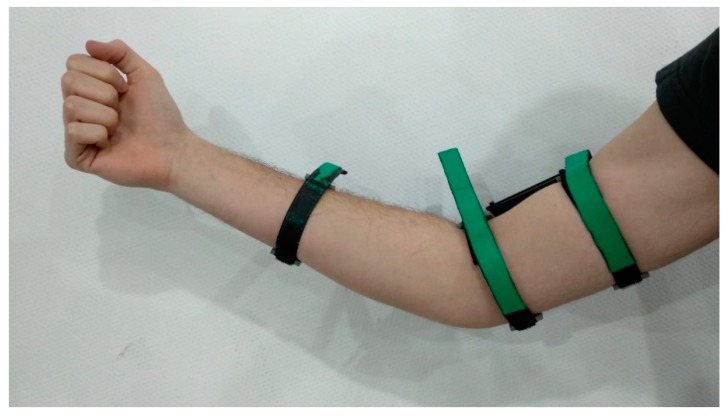
Designed device deployed on a user’s arm. The device is fixed through the three velcro straps.

The set of elements that make up the EXOMedical device are described below. This exoskeleton for the elbow consists of two parts that are attached to the outside of the arm and forearm with a series of velcro straps, as shown in Figure 1. The device has a rotation axis or DOF corresponding to the axis of rotation of the elbow joint.

The structure of the prototype has been built using 3D printing technology since it allows the construction of low-cost prototypes in a short time. Before performing the printing process, the 3D modeling phase has been carried out. This phase has required a long period of time due to the measurement and adjustment of the design. So it is adjustable to a large number of users. Considering the parametric design, it has been possible to design the structural 3D pieces in different sizes to adapt to the measurements of the different patients. Once the structure has been designed, it has been 3D printed with resistant and low-cost material such as PLA (Polylactic Acid). In future versions of EXOMedical, for cases where greater resistance is required, it will be possible to print a new exoskeleton on more durable materials such as ABS, NYLON, or carbon fiber. The motor used in the exoskeleton is a servo motor with a force of 15 kg, which is responsible for assisting the user in the task of exercising the arm. This servo, at the same time, allows making known the position in which the arm is, that is, the opening angle that will be used as an input value in the VR environment. In the same way that happens with the types of plastic, this servo can be equally scalable to get a greater pushing force helping to lift a greater amount of weight. This will be interesting in cases in which the patient has a superior muscular capacity. As can be seen, the device is highly configurable based on the needs and characteristics of the patients.

The load cell in the exoskeleton has the functionality of obtaining the force that the user is exerting at a given moment. In this way, it is possible to know if it is necessary to apply a greater or lesser amount of assistance force with the motor for the realization of the movement, or the lifting of loads, as well as to measure the performance and evolution throughout the realization of the exercises. Specifically, the load cell used is based on a Gauge type cell that measures the electrical resistance in response to a force that is applied to the device. Generally, the element that measures that resistance, also known as a deformation meter, is made up of a very fine wire usually made of copper or aluminum material. This measuring element is based on a grid design so that when pressure is applied to this element, a linear change in resistance occurs. The resistors used in strain gauges are typically 120, 350, and 1000 Ω. The resistances used in each of the load cells have a different sensitivity to the voltage, a variable called “gauge factor”, which is generally used in metal-type strain gauges that are commonly used in most devices, is close to 2.

The load cell model used in this work is the TAL220, industrially used in different fields. This load cell is designed for operating ranges between 2 and 200 kg. Specifically, the one that has been used is the 30 kg load cell since it is the closest assistance value to the average weight that a person can lift in a rehabilitation process. The internal resistance of the TAL220 model is all 1000 Ω.

**Figure 2:**
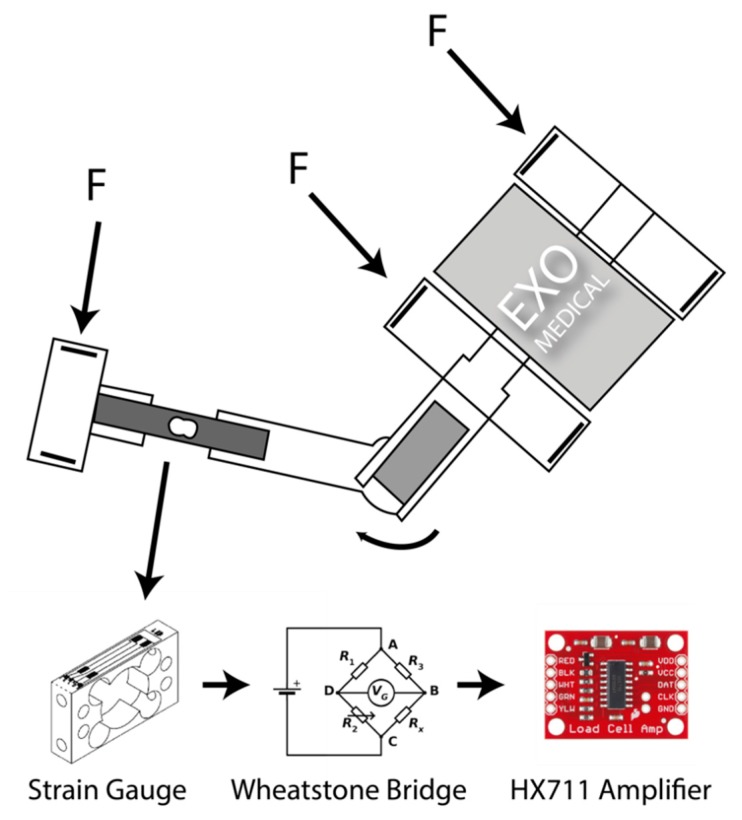
Measurement of the force (F) exerted by the user through the exoskeleton and measured through a Gauge type load cell that is amplified through a Wheatstone bridge.

However, experimentally the following is verified: The variations that occur in a deformation meter are relatively small, of about few millistrain $\left(e\cdot 10^{-3}\right)$. The electrical resistance that provides the magnitude of the force being exerted on the device is very small. Therefore, most of the electronic devices used to digitally measure the value obtained are not adequate, since being so small they do not accurately detect the real value. Therefore, it is necessary to use an electronic device that is capable of accurately measuring the values and changes that occur in the electrical resistance and converting that unit into something that we can measure with a unit of mass (g). For this purpose, an HX711 model amplifier module will be used, the operation of which is based on the use of 4 resistors with a known voltage, forming the “Wheatstone bridge” as shown in Figure 2.

If Vin is a voltage that is constant and known and the resulting Vout is measured as follows *R*1/*R*2 = *R*3/*R*4 it follows that Vout is 0, however, if there is a variation in one of the resistors, the Vout it will be affected, governed by the following Equation (Ohm’s law) (1):

(1) $$V_{out}=\;\left[\left(R3/\left(R3+R4\right)-R2/\left(R1+R2\right)\right)\right]\times V_{in}$$

**Figure 3:**
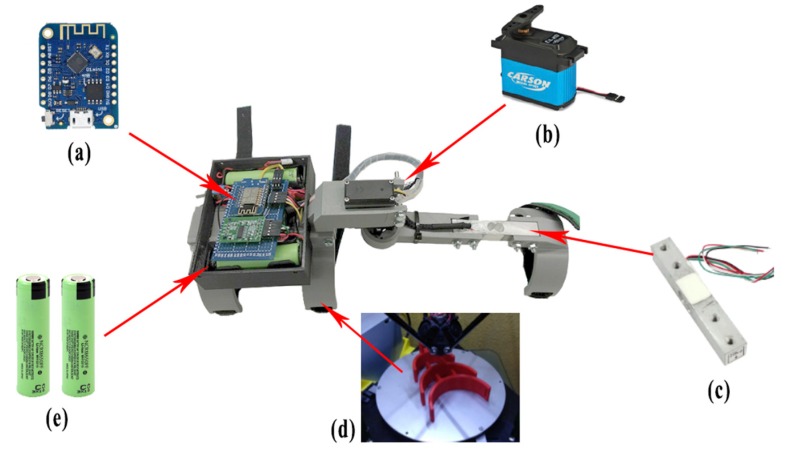
Components of the exoskeleton of a one degree of freedom (DOF) EXOMedical: (**a**) ESP8266 Wireless microcontroller; (**b**) 15 kg servo motor; (**c**) Load cell up to 25 kg; (**d**) 3D printed parts in PLA; (**e**) Two 18,650 lithium batteries of 3.7v 3400 mah each.

If one of the resistors in the Wheatstone bridge is replaced, the resulting value in Vout can easily be measured and the force applied to the load cell can be obtained. On the other hand, the microcontroller used is an ESP8266. It is a microcontroller that contains 8 digital outputs and an analog input. It also has the ability to connect to the data network through WIFI technology and thus be able to send and receive data from the software architecture and VR environment without cables. It is also a low-cost device that allows its incorporation in a large number of Internet of Things (IoT) projects today [35,36]. So that the system is portable and does not depend on an external power source, two 18,650 lithium batteries with a capacity of 3400 mah have been incorporated into the final system, which allows the user a minimum autonomy to perform exercises in a full rehabilitation session. This device can also be used to carry out movements and weight loads in the domestic or work environment as part of rehabilitation therapy and reintegration into everyday life. It should be noted that the final device has a very low cost, with the price of all components being less than €100. This allows becoming an affordable tool for any patient or rehabilitation center that needs it. Figure 3 shows the parts that make up the final device.

### 3.2. Context-Aware Architecture

The important boom that has emerged in recent years in areas such as the Internet of Things and specifically in the so-called Wearable Internet of Things (WIoT) for the collection of physiological and medical data, has led to the application of new paradigms. New paradigms such as medical distributed computing that combine medical IoT devices and sensors to generate from the home environment, medical telemonitoring systems, and remote telecare. The existing WIoT architectures are highly centralized. During the last decades, cloud computing has been the technology base used to process and store data in the cloud instead of using the devices with limited resources that are composing the IoT environment. Cloud infrastructures facilitate the processing, storage, visualization, and real-time analysis of WIoT medical data. However, that cloud-based IoT health system is finding many barriers to handling the large health data that IoT currently generates. Edge computing (EoT) is a new technology that seeks to act as a service-oriented intermediate layer between the IoT health system and cloud computing. Edge computing is a solution that is in full expansion and offers efficient solutions to meet the important demands that telemedicine systems currently have. The objective is to increase the capacities available on the network, such as computing and storage capacity. These operations are performed on the edge of IoT-based systems, near data sources. Consequently, it moves calculations, storage and service provision from clouds to local peripheral devices, such as smartphones, gateways or smart routers, local wireless devices. These edge devices improve connectivity and allow to increase speed and data transfer for a faster and more efficient response.

**Figure 4:**
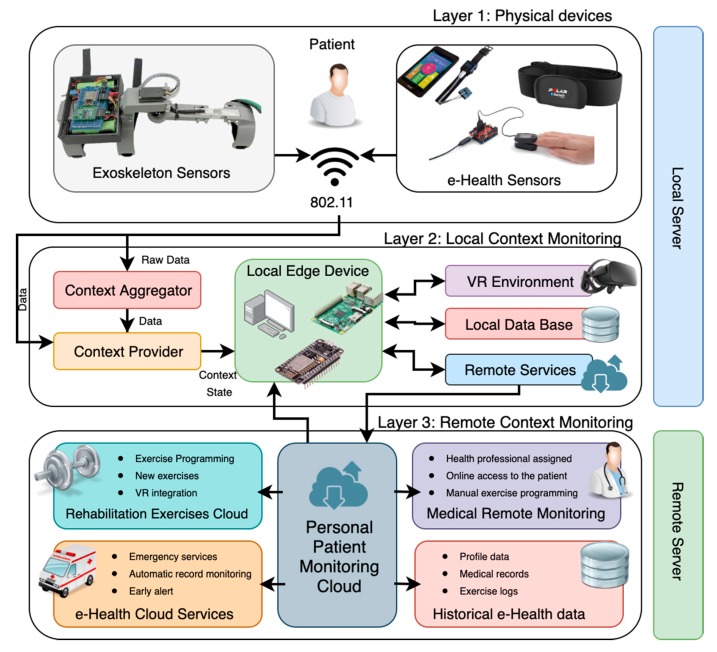
General diagram of the Context-Aware architecture for the EXOMedical system.

Context-Aware systems for current e-health environments are very focused on patient monitoring and follow-up. Specifically, these environments have been highly developed to perform a specific assistance task that helps the elderly patient. In addition, these are very centralized systems, especially focused on traditional Cloud environments. The disadvantages of these systems are the inability to handle large data due to their local structure, the lack of ability to generalize to other patients and other diseases, and lack of context-awareness. To all this, it is necessary to highlight the fact that there are hardly any works of Context-Aware systems focused on the aspect of rehabilitation of tasks and physical pathologies as the case of this work. Figure 4 shows the Context-Aware architecture based on EoT environments for the integration of exoskeletons and rehabilitation of patients through VR environments that are divided into three layers.

*Layer 1: Physical devices:* This is the physical layer that binds both the exoskeleton sensors (load cell, engine position, battery level) and the possible medical sensors that can be added to the system and that can be used to monitor added medical parameters. As in the case of the pulse sensor or the ECG sensor that can emit valuable information about the patient’s physical condition while performing a rehabilitation exercise. All these devices, including the exoskeleton, have an 802.11 WiFi connection through which the data can reach the platform wirelessly.

*Layer 2: Local Context Monitoring*: In this layer, which is deployed on the computer system at the local edge, there are the Context Provider and Context Aggregator modules that are responsible for managing the data generated by layer 1 (sensor system). There are also virtual reality blocks, the local database and the set of functionalities and remote services.

- *Context Aggregator*: This module is responsible for combining all simple contexts into a single Context State through the use of a context model [37]. This element is key when relating all the context data that alone cannot provide any data or be misinterpreted. Therefore, past and present contextual information must be added when making a correct classification of the situation. After performing this contextual aggregation, the information generated is sent to the Context Manager System module for each specific user.
- *Context Provider*: It is a service that is the main source for generating contexts. The Context Aggregator module transfers the low-level raw data generated by the different sensors to the Context Providers modules. These modules, in turn, are responsible for applying well-known techniques on this data to generate an elementary context from low-level data. For example, by applying pattern recognition techniques, it is possible to determine if a patient is correctly performing an exercise or is exhausted and therefore the exercise is being useless for rehabilitation.
- *VR Environment*: This module will be responsible for managing communication with the virtual reality simulation environment. It will act as coordination to send the exoskeleton data to the simulator and thus be able to make a real integration with the 3D environment. He will also be in charge of receiving back the values of the different tests that measure the performance and therefore the progress of the patient throughout the treatment. It will also be the key when generating the new scenarios and configure parameters generated by the system as the user advances or correct certain parameters that are determined to be unhelpful in case of detecting a lack of progress or regression.
- *Local DataBase*: It is a module responsible for managing the local database of the system that will store both the values of both the sensors deployed in layer 1 and the Context State generated by the Context Aggregator as well as all the results and evaluations produced during the VR exercises and simulations. This database has a double functionality. On the one hand, to perform a local persistence of the data to avoid loss or alteration of the data. On the other hand, a supporting function at the end of the computing tasks to identify patterns or anomalous situations in data.
- *Remote Services*: It is a module in charge of managing the remote services offered by Cloud architecture. Communications, data steps, and events are managed by this module, which will be in charge, for example, of managing and issuing the necessary alerts in the event that the system detects an abnormal situation in medical sensors. It will also be responsible for access services and medical records or request new exercises for a patient.

*Layer 3: Remote Context Monitoring*: This last layer is located in an external and environmental Cloud to the other two layers. Its mission will be to manage and integrate the remote services for monitoring the context generated in the previous layers. This layer is managed by the central Personal Patient Monitoring Cloud module that is responsible for managing the rest of the modules and services of the system. It is a personal cloud that will be instantiated by each of the users so that all users have a private and secure cloud space. Among the modules deployed are Rehabilitation Exercises Cloud, e-Health Cloud Services, Medical Remote Monitoring, and Historical e-Health data:

- *Medical Remote Monitoring*: It is a module that will be responsible for providing the assigned medical professional with a set of functions for the proper management of the patient. Among these functions are online access to patient data, statistics, and advances. Reassignment or referral to other medical professionals to perform other treatments or alternative diagnoses. The assignment of new exercises for the patient or the modification of the configurations of certain exercises (modification of the number of repetitions, modification of the maximum weight or minimum weight, etc.).
- *Historical e-Health data*: This module will manage all the data generated in the previous layers and that can be accessed by authorized medical professionals, as well as by the rest of the modules. This is a copy of the local database, which will also have the patient’s personal information and all his previous history.
- *e-Health Cloud Services*: This is the module in charge of the automatic management of the data generated and emitted by the system. This module will have the necessary internal tools and processes for the early detection of anomalies in medical data. As well as the constant monitoring of rehabilitation exercises performed by the user. It also has an automatic emergency called system if necessary.
- *Rehabilitation Exercises Cloud*: The system will have a large set of exercises and centralized configurations for performing rehabilitation exercises by patients. It will be possible to register new exercises, manage integration with the VR environment, and schedule exercise sessions that will be sent to the Local Context Monitoring environments of the different patients.

### 3.3. Virtual Reality System

The virtual reality system that integrates the EXOMedical exoskeleton is based on the Unity 3D environment engine [38]. Integrating VR technology into rehabilitation treatment is based on three key aspects: Repeat exercises, feedback, and patient motivation. The improvement and evolution of motor function will depend on the correct repetition of the exercises proposed. The VR technology-based training system can provide additional stimulation to promote motor learning and maintain those abilities over time. The interactive experience facilitated by VR environments makes patients naturally focused on training and evolved in a faster and more efficient way. There are five basic principles when designing VR environments for physical rehabilitation exercises:

- The content of the exercises or games must be attractive and reasonably logical to capture the patient’s motivation.
- Positive feedback should be generated, encouraging rewards that help patients feel they are improving.
- Rehabilitation tasks and exercises should be integrated into exercises and games in an appropriate way and advised by an expert.
- Difficulty levels and settings must be designed to be applied to different patients and different stages of rehabilitation.
- Information on performance and evolution of patient training should be generated in real-time so that the system is able to assess its evolution and adapt to the context to generate new exercises or modify levels and settings.

**Figure 5:**
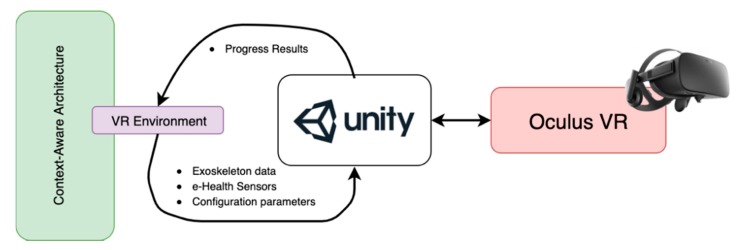
Main components of the virtual reality environment based on the Unity 3D engine and the Oculus system.

The EXOMedical device is integrated with the Oculus VR device [39] through different virtual scenarios in which the patient must perform rehabilitation exercises. The block diagram in Figure 5 represents the main components of the configuration for the VR environment and the Context-Aware architecture.

**Figure 6:**
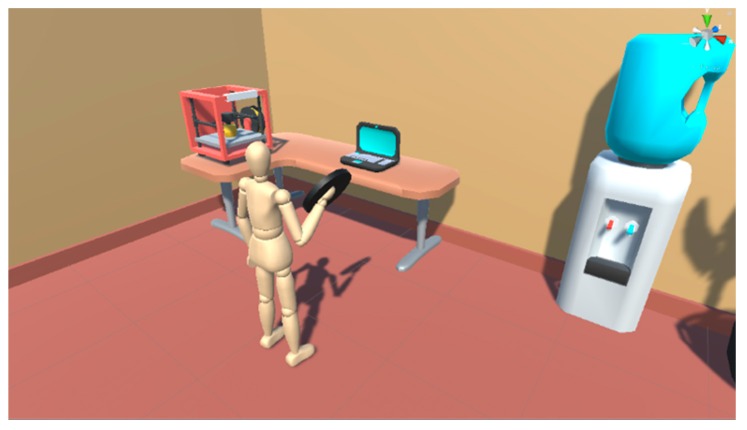
The capture of one of the exercises performed through the Unity 3D environment to be reproduced in the Virtual Reality environment. The user must move the disk and deposit it on the table as part of an initiation exercise in the system.

The patient who is performing an exercise in the system, receives prior instructions on each of the exercises, visualizing through examples, the movements to be performed and the objective of the exercise or game. It also receives a set of visual and acoustic feedback that provides information about everything that happens in the VR environment. The user visualizes an avatar that represents their movements in the virtual environment and is able to recognize if they are performing the task correctly. In addition, visual or acoustic feedback can help patients understand the exact moments of the beginning and end of each exercise. This data is one of the most important because it provides the level of performance of a task, as well as the level of awareness of the patient about their evolution for the required exercise. The system can automatically modify some of the task parameters according to the real-time analysis provided by the Unity control unit. It will be possible to modulate each difficulty level of the exercise or the maximum time to complete the exercise. The environment will provide a reward in the form of scoring or unlocking new levels as the user’s skill progresses. All these data can also be modified by the medical expert in charge of the patient remotely, as well as visualize the results in real-time. The expert can monitor that the parameters generated automatically do not pose a danger to the patient. For example, to prevent the system from generating a very high simulated weight or a maximum unreachable time. Figure 6 shows some examples of exercises included in the system and which are simulated in the Unity VR environment through the Oculus device. Specifically, one of the first exercises designed for the user to learn to use the virtual environment by moving the weight from one point to another is shown.

## 4. Experimental Results

With the aim of validating the system in a real environment, an experiment with 5 patients has been carried out over a period of one month. The study has been carried out due to the collaboration of a specialized physiotherapy rehabilitation clinic. All patients have voluntarily agreed to perform this experiment and have been duly informed. All procedures performed in studies involving human participants were in accordance with the ethical standards of the 1964 Helsinki declaration. The work has been approved by the ethical committee of the Expert Systems and Applications Laboratory Research Group (approval code SA-2201/1). Four of the volunteers (three men between the ages of 23 and 47 and two women between 29 and 37) had previously been diagnosed with lateral epicondylitis of the elbow (or tennis elbow), while one of them was healthy (control patient). The study has been carried out over a month, where volunteers have held four sessions, the first session on day 1 of the study, the second at 7 days, the third at 14 days and the fourth at 30 days.

**Figure 7:**
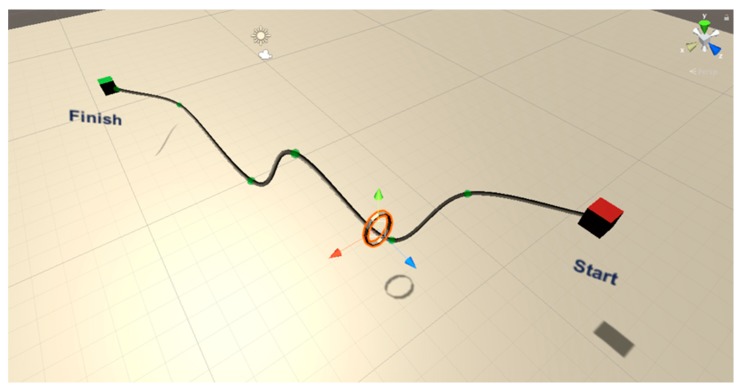
Virtual Reality version of the Buzz Wire Game performed in the case of study. In the center, it is possible to observe the ring (in orange) that the user must transport from the starting point (in red) to the endpoint (in green).

The activities carried out in each session were simple exercises in the Virtual Reality environment, with different configuration parameters. Specifically, the patients carried out a Virtual Reality version of the classic Buzz Wire Game as shown in Figure 7. It is a game where the user must move a ring through a cable from the starting point to the endpoint. This ring should not touch the cable at any time while moving from one point to another.

**Figure 8:**
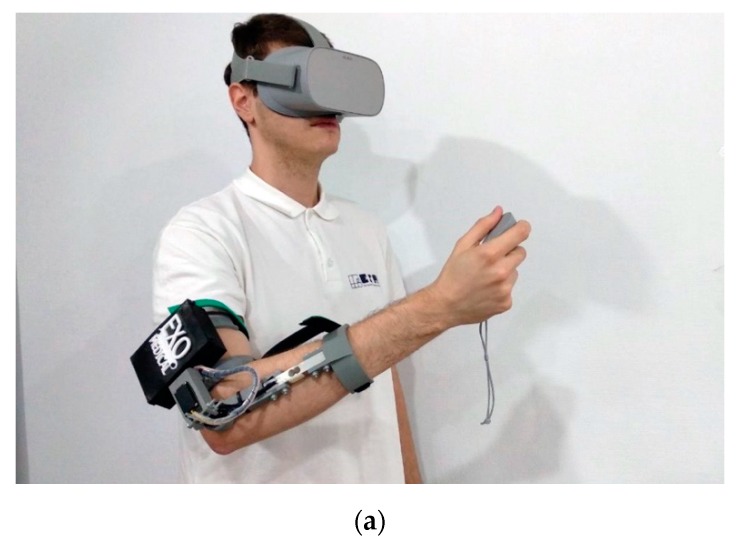

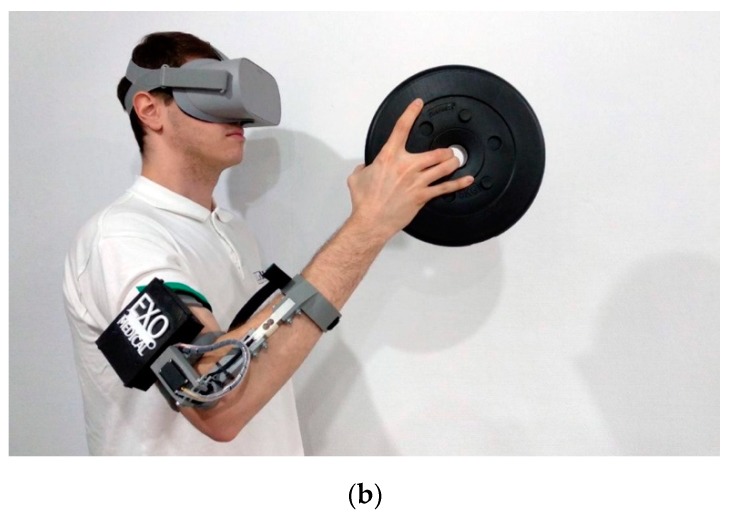
Patient performing a rehabilitation exercise through the VR environment and the exoskeleton for the elbow: (**a**) The user performing exercises with simulated weight through the control of the VR device; (**b**) The user performing exercises with real weight and exoskeleton assistance.

During each of the case study sessions, participants performed 20 activities of this game, progressively increasing the difficulty. Specifically, with each new exercise, the number of cable curves is increased, the internal diameter of the ring decreases and the estimated time to complete the exercise is reduced. As control variables, the positions, angle and time to perform each of the exercises have been analyzed. Of the 20 activities, 10 have been designed to be performed with simulated weight and the other 10 exercises with real weight. Figure 8 shows a user who performed a test on the system with the EXOMedical exoskeleton and the Oculus VR equipment.

The results achieved by the patients involved in the case study can be observed in Table 1, though the number of collisions detected between the ring and the cable. As can be seen, in the initial sessions, the number of average collisions detected is greater in the initial sessions than in the last session. Similarly, the collisions detected by the VR system are greater in cases where the weight used was real, compared to cases in which the weight was simulated (through engine effort). In the case of the results obtained by the control patient, it is observed that despite not having an elbow injury, the difficulty of the exercise has caused that he makes some mistakes while performing the exercises with both real and simulated weight.

**Figure 9:**
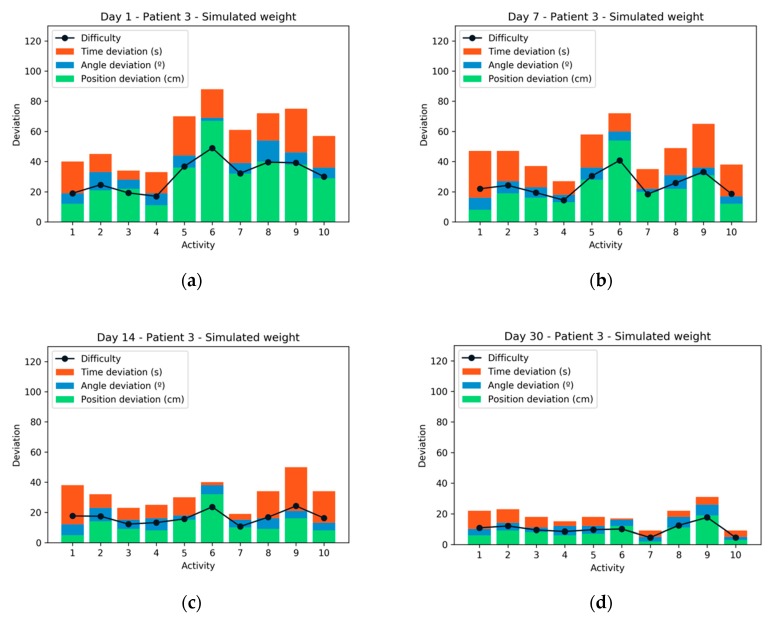
Performance and difficulty experienced by patient 3 during the 4 sessions with simulated weight: (**a**) Session 1; (**b**) Session 2; (**c**) Session 3; (**d**) Session 4.

By individually analyzing one of the patients during the four sessions, it is possible to observe the evolution in their performance with the passing of the sessions. As can be seen in Figure 9, the first two sessions were more difficult for patient number 3 than the last two sessions where the deviation in both time and angle and position was clearly lower. The deviation value represents the distance from the same exercise performed perfectly. This value is measured at three points: Distance, angle, and time with respect to the perfect path. The value of the difficulty is calculated based on Equation (2) where the deviation of time has a weight less than the deviation of the other two parameters.

(2) $$\begin{array}{c} Difficulty=\left[\left(\sum \mathrm{Position}\;\mathrm{deviation}+\sum \mathrm{Angle}\;\mathrm{deviation}\right)*0.6\right]\\ +\left(\sum \mathrm{Time}\;\mathrm{deviation}*0.4\right) \end{array}$$

**Figure 10:**
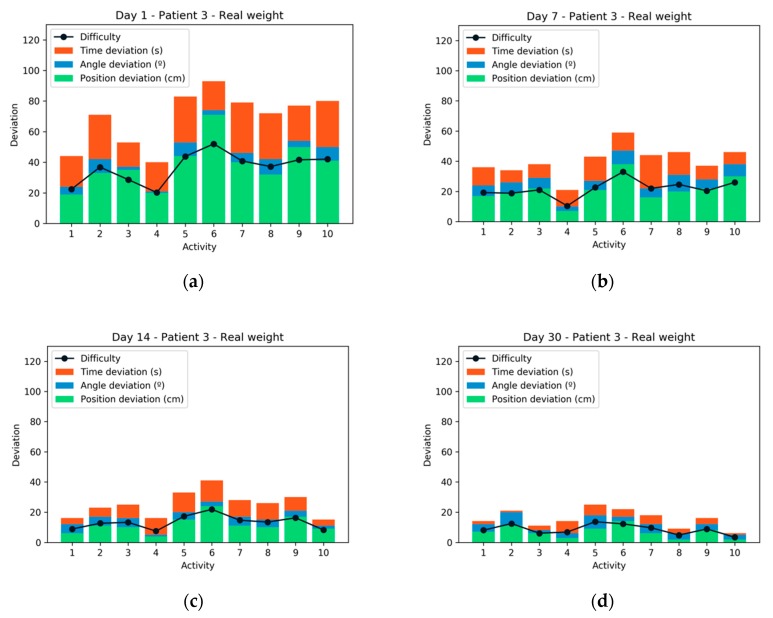
Performance and difficulty experienced by patient 3 during the 4 sessions with real weight: (**a**) Session 1; (**b**) Session 2; (**c**) Session 3; (**d**) Session 4.

If patient 3 is analyzed for the same activities but performed with the real weight, it is possible to observe how the average performance was worse than in the case with simulated weight (as shown in Figure 10). In the final sessions, the patient achieved a high-performance due to the strengthening and practice with the same exercises. After each training session, patients were consulted through a paper test about their experience. They all agreed that simulated weight training was more comfortable and useful for performing their exercises. There was also unanimity when preferring the virtual reality-based system, to the traditional rehabilitation system.

**Figure 11:**
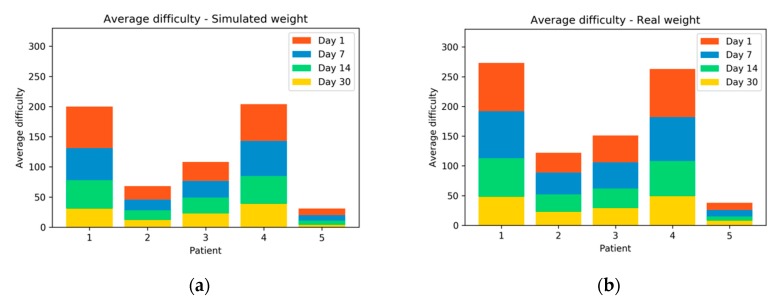
The average difficulty of each patient in the four sessions performed; (**a**) With simulated weight; (**b**) With real weight.

Figure 11 compares the results obtained in difficulty for each patient in each of the sessions. As can be seen, the average difficulty in exercises performed with real weight is greater than exercises performed with simulated weight. It can also be seen how patient 2 has very high results with simulated weight. Very close to the results of the control patient (patient 5). The patient who experienced the greatest difficulty is patient 1, both with real weight and with simulated weight.

## 5. Conclusions

This article presents the design of a training system for the rehabilitation of the elbow through a low-cost exoskeleton that is integrated with a virtual reality system. On this system, an architecture based on Context-Aware has been designed for intelligent management, the detection, and monitoring of the advances made by a patient in the system. The system has the functions of evaluating the rehabilitation, the advances, and all the data generated, with the aim of inferring knowledge that allows helping in its rehabilitation. Virtual reality technology is integrated into patient rehabilitation training in the form of interactive activities and games that generate a positive and motivating effect. These games and exercises are designed in such a way that the patient knows the state in which the exercise is, as well as its evolution and performance. Joint rehabilitation is achieved through fun repetitive training tasks where levels of difficulty are gradually increasing. In addition, through the Context-Aware architecture designed, a layer of intelligence is added to the system that allows, among other things, to track the automatic patient that, depending on the context data, allows new levels to be generated for their exercises or to detect possible anomalies or health problems.

Compared to other rehabilitation systems based on exoskeletons and similar VR environments, this system has differential advantages. For example, in data acquisition, compared to other existing systems, the designed exoskeleton allows it to be installed in a simple way. It has a low cost and without cables that prevent a natural movement of the arm. In the aspect of the design of the exercises, the mechanisms and activities of this system are patterned according to the training specifications that are most suitable for proper rehabilitation. In addition, the exercises and games are extensible and easily expandable, due to the powerful engine designed by Unity3D and Oculus. It is also important to highlight how the rehabilitation plan will be generated automatically for the Context-Aware system and the observations of the medical expert in charge of the rehabilitation. This represents an important advance with respect to the systems in which only the expert could modify the parameters of the exercises. Considering the important database generated, and the different cases treated by the system, as new workouts are generated, the system will further refine its diagnoses and configurations. It should also be noted that the contents of the exercises and rehabilitation games can be loaded remotely by the system, being possible to add more elements to daily activities and generating a new sense of motivation about the patient. This allows the system to enrich the content of the activities and adapt them to the advances and performances of the patients. It is also possible to include more rehabilitation techniques in the future since the architecture is designed to open enough to do so. It is a connected device, aligned with the new technologies and paradigms based on IoT that allow managing data remotely, being accessible from anywhere in the world. In addition, the platform will allow not only the rehabilitation of patients but constant monitoring and follow-up of their medical parameters, being a very useful tool to be deployed in domestic environments.

As a line of future work, we will study how to expand the exoskeleton to allow the rehabilitation of more arm joints and even the leg or neck. It will also be interesting to study exoskeletons for the rehabilitation of hands and phalanges of the fingers. In a future version of the system, a more friendly backend interface will be included so that any medical expert can configure the system parameters in a simpler way. Another line of future research will be to conduct a case study with a higher number of patients and with different medical problems to compare the evolution according to the pathology.

## Figures and Tables

**Table 1:** The number of collisions detected in each session by the different patients, divided between exercises with real and simulated weight.

Patient/weight		Session 1	Session 2	Session 3	Session 4
**1**	Simulated	47	31	28	9
	Real	58	50	39	18
**2**	Simulated	68	55	46	19
	Real	137	112	64	27
**3**	Simulated	90	75	66	18
	Real	147	122	98	34
**4**	Simulated	60	47	20	11
	Real	67	40	32	21
**5 ^1^**	Simulated	6	7	6	4
	Real	11	9	9	6

^1^ Control patient.
